# Supplementary material for: A Systematic Investigation on the Influence of Intumescent Flame Retardants on the Properties of Ethylene Vinyl Acetate (EVA)/Liner Low Density Polyethylene (LLDPE) Blends
Source: Molecules. 2023 Jan 19;28(3):1023. doi: 10.3390/molecules28031023 (PMC9921819; doi:10.3390/molecules28031023)
Supplement: Supplementary file 1 [file molecules-28-01023-s001.zip › molecules-2077306-supplementary.pdf]

A systematic investigation on the influence of intumescent flame retardants on the properties of ethylene vinyl acetate (EVA)/liner low density polyethylene (LLDPE) blends

Eid Alosime[1]* and Ahmed A. Basfar[2]
[1] King Abdulaziz City for Science and Technology, P.O. Box 6086, Riyadh 11442, Saudi Arabia.
[2] King Saud University, College of Engineering, Mechanical Engineering Department, P.O. Box 145111, Riyadh 4545, Saudi Arabia.
Corresponding to: alosimi@kacst.edu.sa

Table S1. EVA/EBM/CLNA-8400/120phr MH flame retardant formulations as a function of Tafmer 805 content.

Content/Property	AP-647	AP-654	AP-655	AP-656	AP-657
EVA	85	80	75	70	65
LLDPE 118W	-	-	-	-	-
Tafmer 805	-	5	10	15	20
CLNA-8400	6	6	6	6	6
EBM	9	9	9	9	9
MH	120	120	120	120	120
Carbon black 550	6.0	6.0	6.0	6.0	6.0
Naugard Q	1.5	1.5	1.5	1.5	1.5
Tensile strength (MPa) at RT	13.0±0.2	13.3±0.2	13.3±0.3	13.9±0.4	14.1±0.4
Elongation at break (%) at RT	208±13	229±12	220±16	222±11	228±12
Tensile strength (MPa) after thermal aging at 100ºC	12.5±0.2	13.1±0.3	13.3±0.1	13.2±0.1	13.3±0.2
Retention of tensile strength (%) after thermal aging at 100ºC	96.1	98.5	100	95.0	94.3
Elongation at break (%) after thermal aging at 100 ºC	201±12	213±8	219±17	229±9	221±4
Retention of elongation at break (%) after thermal aging at 100 ºC	96.6	93	99.5	103.2	96.9
LOI (%)	30.0	30.5	30.0	30.0	30.0

Table S2. EVA/120 phr MH formulations as a function of EBM content.

Content/Property	AP-586	AP-638	AP-639	AP-640	AP-641
EVA	85	91	88	85	80
LLDPE 118W	15	-	-	-	-
EXACT 8201	-	-	-	-	-
CLNA-8400	-	-	-	-	-
EBM	-	9	12	15	20
MH	120	120	120	120	120
Carbon black 550	6.0	6.0	6.0	6.0	6.0
Naugard Q	1.5	1.5	1.5	1.5	1.5
Tensile strength (MPa) at RT	11.3±0.3	14.2±0.4	14.3±0.1	14.6±0.2	14.5±0.1
Elongation at break (%) at RT	177±10	225±8	214±13	216±15	194±15
Tensile strength (MPa) after thermal aging at 100ºC	12.6±0.2	12.7±0.3	13.5±0.2	13.6±0.3	13.3±0.3
Retention of tensile strength (%) after thermal aging at 100ºC	111.5	89.4	94.4	93.2	91.7
Elongation at break (%) after thermal aging at 100 ºC	179±8	219±5	219±6	212±6	192±8
Retention of elongation at break (%) after thermal aging at 100 ºC	101.1	97.3	102.3	98.1	98.9
LOI (%)	38.0	31.5	31.0	30.0	31.0

Figure S1. The morphology of the prepared composites was studied by scanning electron microscopy (SEM).

Figure S1 displays SEM micrographs of the EVA/LLDPE 118W and selected corresponding composites containing 120 phr flame retardants (MH+RP).

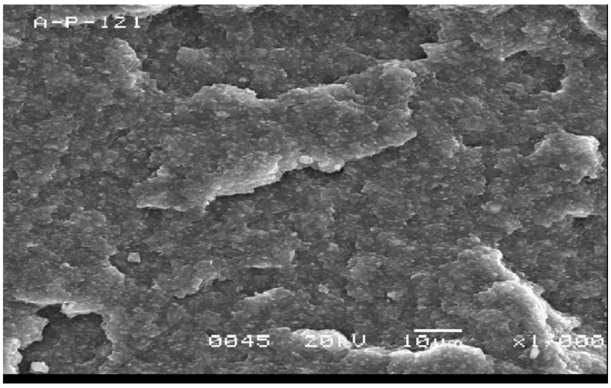

LDPE:EVA = 20:80
with 120 phr magnesium hydroxide (MH) (AP-587)

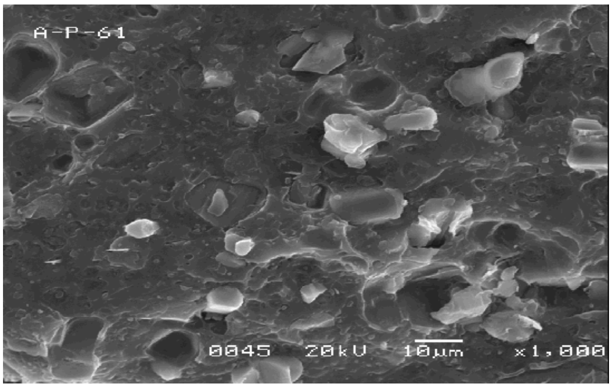

LDPE:EVA = 20:80
with 120 phr magnesium hydroxide + red phosphorus (AP-663)

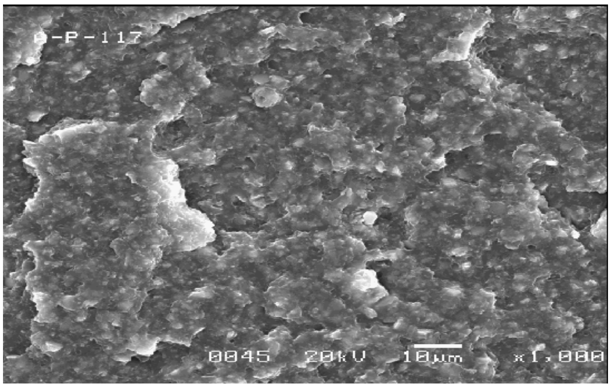

LDPE:EVA = 20:80
with 120 phr magnesium hydroxide + red phosphorus (AP-665)

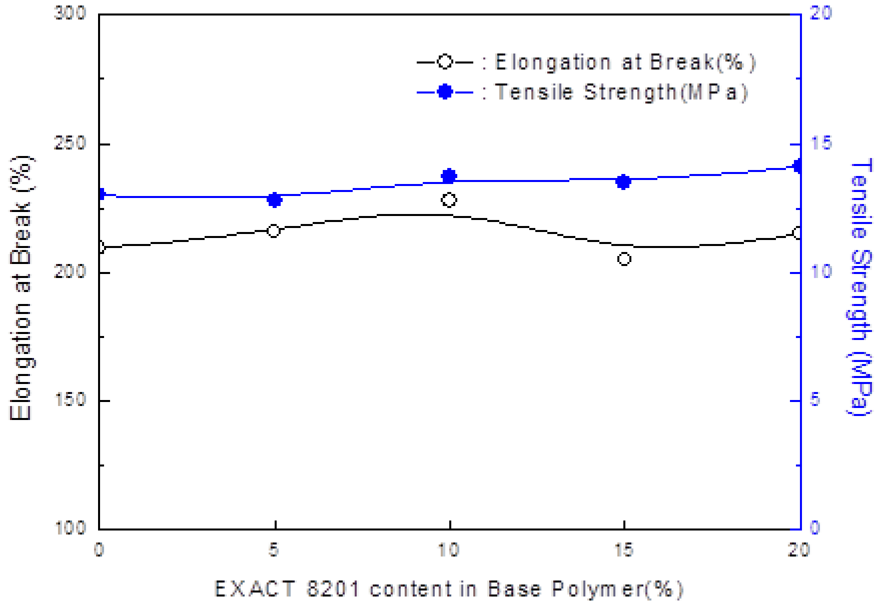

Figure S2. Mechanical properties of EVA/CLNA-8400/EBM/120phr flame retardants (MH) formulations as a function of EXACT 8201 content.
